# Investigation of Twelve Significant Mycotoxin Contamination in Nut-Based Products by the LC–MS/MS Method

**DOI:** 10.3390/metabo12020120

**Published:** 2022-01-26

**Authors:** Buket Er Demirhan, Burak Demirhan

**Affiliations:** Department of Pharmaceutical Basic Sciences, Faculty of Pharmacy, Gazi University, Ankara 06330, Turkey; bdemirhan@gazi.edu.tr

**Keywords:** nut, chocolate, mycotoxin, microbiologic analysis, LC–MS/MS

## Abstract

In this study, a total of 80 peanut butter, hazelnut butter, and chocolate samples were obtained from local markets in Ankara, Turkey. These foods were analyzed for twelve toxicological important mycotoxins, such as aflatoxin B1 (AFB1), aflatoxin B2 (AFB2), aflatoxin G1 (AFG1), and aflatoxin G2 (AFG2); fumonisin B1 (FB1) and fumonisin B2 (FB2); ochratoxin A (OTA); sterigmatocystin (STE); deoxynivalenol (DON); zearalenone (ZON); T-2 toxin (T2); and HT-2 toxin (HT2) by the LC–MS/MS multi-mycotoxin method. In addition to this analysis, the presence of total aerobic mesophilic bacteria was investigated in the samples. The samples were analyzed microbiologically using standard procedures. Finally, the minimum and maximum levels of AFB1, AFB2, AFG1, FB2, OTA, STE, DON, ZON, T2, and HT2 in the samples were found to be 0.04–27.37 µg/kg, 0.06–6.19 µg/kg, 0.14–0.40 µg/kg, 2.73–2.93 µg/kg, 0.01–37.26 µg/kg, 0.19–2.25 µg/kg, 11.81–42.09 µg/kg, 0.03–7.57 µg/kg, 1.41–2.54 µg/kg, and 6.94–7.43 µg/kg, respectively. AFG2 and FB1 were not detected in any of the samples. The most frequently detected mycotoxins in analyzed samples were OTA (78.75%) and AFB1 (75%). In addition, total aerobic mesophilic bacteria were isolated from 53.75% of samples. Some of the tested food samples contained mycotoxins above the Turkish Food Codex maximum limit.

## 1. Introduction

Today, people are exposed to many different chemical contaminants from different sources, and food is one of the important reservoirs for this exposure [1]. Foods contaminated with pathogens, harmful microorganisms, or chemicals can cause the formation of more than 200 diseases in humans [2]. Under favorable environmental conditions during cultivation, processing, and storage, fungi can grow and nuts can become contaminated with these fungi [3]. Mycotoxins, the secondary metabolite produced by some fungi, are a common food contaminant that causes serious health problems, such as poisoning in animals and humans [4]. Since mycotoxins negatively affect the quality and safety of agricultural products; they can cause yield and economic losses worldwide. [4,5]. Some mycotoxins, such as aflatoxins, ochratoxin, deoxynivalenol, and zearalenone produced by Aspergillus and Penicillium genera, and fumonisins B produced mainly by Fusarium species, are considered to be of great economic and toxicological importance for cereal crops [4]. Currently, the most important mycotoxins, in terms of food safety and regulations, are considered to be aflatoxins, ochratoxin A, deoxynivalenol, fumonisins, zearalenone, ergot alkaloids, T-2 toxin, HT-2 toxin, patulin, and citrinin [6].

Exposure of humans to these contaminants can occur through the consumption of agricultural food products, such as contaminated grains, nuts, and dried fruits, or indirectly through the consumption of animal-derived foods, such as contaminated milk, meat, and eggs. [7,8]. Nuts such as hazelnuts, almonds, walnuts, pine nuts, pistachio, and cashew nuts contain proteins, oils, unsaturated fat, vitamins, and essential minerals [1,9]. Therefore, nuts are recommended components of the daily diet [1]. Cereals, various types of nuts, and products are the main sources of exposure to mycotoxins [6]. A diet containing high mycotoxin levels can cause acute and/or chronic adverse health effects in animals and humans. Mycotoxins can affect many organs in the human body, such as the liver and kidney, and many systems, such as the endocrine system, the immune system, and the nervous system [10]. The consumption of crops contaminated with mycotoxins that cause acute or chronic health problems is very important for consumers in terms of food safety and public health [11]. Mycotoxins, which are frequently detected from foods and feeds, pose a significant risk due to their hepato-toxic, mutagenic, nephrotoxic, immunosuppressive, and carcinogenic effects as a result of human and animal exposure to these mycotoxins [12].

Peanuts (*Arachis hypogaea* L.) are one of the most important and popular oilseed crops and snack foods in the world. These are often contaminated with several microorganisms from various environmental sources in the post-harvest processes of the traditional harvest [13]. In addition, the safety of peanuts and peanut-derived products should be considered throughout production for the risk of foodborne illness [14]. Nuts such as hazelnuts are rich in protein and unsaturated fat and are resistant to microorganism growth because they contain low amounts of water. However, fungal contamination of nuts can occur during cultivation, processing, or storage in environmental conditions suitable for fungal growth. Therefore, people may be exposed to mycotoxins, especially aflatoxins, as a result of the consumption of hazelnuts that are processed and stored under inappropriate conditions [1].

Although chocolate with low water activity is generally accepted as a microbiologi-cally stable product, the water activity in chocolates is not low enough to inhibit the growth of molds. The quality of chocolate depends on environmental, agricultural, and technological factors. Microorganisms that provide fermentation are among these factors and it is stated that they play an important role in the development of the sensory proper-ties of chocolate. The presence of another group of microorganisms during microbial fer-mentation is an important factor affecting the production of a good quality product. It is stated that one of the most common molds in cocoa beans is Aspergillus spp. [15].

The aim of the present study was to investigate mycotoxin presence and total aerobic mesophilic bacteria in peanut, hazelnut, and chocolate obtained by Ankara local markets in Turkey.

## 2. Results

Toxicological twelve mycotoxins (AFB1, AFB2, AFG1, AFG2; FB1, FB2, OTA, STE, DON, ZON, T-2 toxin, and HT-2 toxin) were analyzed in 80 food samples, including peanut butter, hazelnut butter, and chocolate, by the LC–MS/MS method. These samples were purchased from Ankara, Turkey.

According to the Turkish Food Codex (TFC), the maximum levels for AFB1 and total aflatoxin (B1 + B2 + G1 + G2) in hazelnuts and their processed products are 5 μg/kg and 10 μg/kg, respectively [16]. Mycotoxin data of chocolate samples are given in Table 1. No sample exceeding the maximum level determined for AFB1 was detected in chocolate samples. It is statistically significant that the mean OTA value of brand A is higher than that of brand B (*p* < 0.05). The difference between the mean values of the two brands for DON mycotoxin was not statistically significant (*p* > 0.05). AFB2, AFG2, FB1, FB2, T2, and HT2 mycotoxins were not detected in chocolate samples. AFB1, AFG1, ZON, and STE mycotoxins were detected only in samples of brand A.

In the TFC, the maximum levels for AFB1 and total aflatoxin (B1 + B2 + G1 + G2) in hazelnuts and their processed products are 5 μg/kg and 10 μg/kg, respectively [16]. Data on the mycotoxin contents of hazelnut butter are given in Table 2. No sample exceeding the maximum level determined for AFB1 was detected in hazelnut samples. The difference between the C and D brands for AFB1, AFB2, AFG1, OTA, STE, T2, and ZON mycotoxins was not statistically significant (*p* > 0.05). For HT2 mycotoxin, the difference between the two brands was statistically significant (*p* < 0.05). AFG2, FB1, FB2, and DON mycotoxins were not detected in the hazelnut butter samples of the two brands.

According to the TFC, the maximum levels for AFB1 and total aflatoxin (B1 + B2 + G1 + G2) in peanuts and their processed products are 5 μg/kg and 10 μg/kg, respectively [16]. The number of peanut butter samples exceeding the maximum AFB1 and total aflatoxin amount specified in TFC was determined as 21 and 12, respectively. Mycotoxin data of peanut butter are given in Table 3. In peanut samples, the average AFB1 values of E, F, and G brands were higher than the maximum AFB1 value specified in the TFC. Three samples of E brand, all samples of F brand, and eight samples of G brand were found to be higher than the AFB1 maximum level. The fact that the values of these three brands are higher than the limit specified in the TFC is very important for both consumers and producers in terms of public health and food safety. The difference between the AFB1 and AFB2 mean values of the F, G, and H brands was statistically significant (*p* < 0.05). The brand with the highest AFB1 and AFB2 mean values are F, followed by G, E, and H brands. H brand has the lowest AFB1 mean value, and the difference between H brand and F and G brands is statistically significant (*p* < 0.05). When the difference between the mean values of OTA mycotoxin is examined, the difference between the brands is not statistically significant (*p* > 0.05). STE mycotoxin was detected only in E, F, and G brands. Among these, the difference between E and F brands was found to be statistically significant (*p* < 0.05). The brand with the highest STE mean value is F brands, followed by the G and E brands. AFG2, FB1, T2, DON, and HT2 mycotoxins were not detected in peanut butter samples.

The total number of aerobic mesophilic bacteria is used to give information about the general hygiene and microbiological quality of foods and to determine spoilage in foods [17,18]. The total aerobic mesophilic bacteria (TAMB) counts of nut butter and chocolates are given in Table 4, while the mean ± S.E, as well as the min and max TAMB data of positive samples, are given in Table 5. The difference between E, F, and G brands in terms of TAMB numbers of peanut butter is statistically significant (*p* < 0.05). The number of TAMB is the lowest in E brand, followed by H, G, and F brands, respectively. TAMB growth was observed in 80% of the total peanut butter. In hazelnut butter, TAMB growth was observed in one sample only in D of the two brands. In the C brand, the number of TAMB is less than 10^2^ CFU/g. In chocolates, the difference between the two brands was statistically significant (*p* < 0.05). The mean TAMB count of A brand was found to be higher than B brand.

## 3. Discussion

Mycotoxins, i.e., secondary metabolites of fungi, are important contaminants that affect feed and food quality, and their importance has increased worldwide as they pose a serious risk to animal and human health [1]. It is stated that because fungal species and strains differ in their ability to infect plants, there may be differences between toxigenic fungi and mycotoxins found in these plants and their crops. It is also reported that crop varieties may show different levels of susceptibility or resistance to toxigenic fungal infections [19]. Mycotoxins can contaminate foods and feeds before or after harvest. Identification of mycotoxin-producing fungal species using traditional isolation and culture techniques before mycotoxin formation is important in terms of early detection. Although identification of fungal species is important for early detection, traditional methods for detecting mycotoxin-producing fungi in food and feed are time-consuming and require expertise. Therefore, it is important to develop rapid and reliable techniques for the detection of mycotoxins in foodstuffs [20]. Copetti et al. [21] investigated the presence of aflatoxin B1 (AFB1), B2 (AFB2), G1 (AFG1), and G2 (AFG2), as well as ochratoxin A (OTA), in a total of 125 powdered, bitter, dark, milk, and white chocolate samples from Brazil. They reported that the mean values of OTA, AFB1, AFB2, AFG1, and AFG2 in powder, bitter, dark, milk, and white chocolate samples were between 0.03–0.39 µg/kg, <LOD-0.43 µg/kg, <LOD-0.08 µg/kg, <LOD-0.29 µg/kg, and <LOD-0.01 µg/kg, respectively. They stated that ochratoxin A was the most commonly detected mycotoxin with 98% of chocolate samples. They state that both ochratoxin and aflatoxins must be systematically monitored to ensure safe chocolate consumption for consumers. Naz et al. [22] investigated the total aflatoxin and ochratoxin A contamination by HPLC-FLD in 200 samples of 100 branded and 100 local chocolates in Pakistan, including bitter, dark, milk, and white chocolate. They reported that most of the samples were contaminated with aflatoxins and ochratoxin A, and the total incidence of aflatoxin and ochratoxin A contamination was 83% and 90% for branded products and 91% and 97% for local products, respectively. They noted that local white chocolates and dark chocolates had the highest average aflatoxin (3.35 μg/kg) and ochratoxin A (3.48 μg/kg) levels. Kabak [23] analyzed dark and milk chocolate and chocolate wafers for Aflatoxins and OTA in Turkey by HPLC-FLD. He reported that OTA was the most commonly detected mycotoxin and its value varied between 0.18 and 0.75 µg/kg. It was reported that OTA detected 46.7% in dark chocolate, 22.8% in milk chocolate, and 17.4% in chocolate wafers. Aflatoxins were also detected in 13.3% of dark chocolate, 19.6% of milk chocolate, and 8.7% of chocolate wafers at concentrations ranging from 0.15 to 2.04 μg/kg. Younis et al. [24] analyzed aflatoxin B1, B2, G1, and G2 in 400 peanut butter samples by HPLC and TLC methods. They stated that 64% of the total peanut butter samples contained aflatoxin, and the average value was 43.5 and 41.9 µg/kg by HPLC and TLC methods, respectively. They reported that the incidence rates of aflatoxin B1, B2, G1, and G2 in positive peanut butter samples were 85%, 3%, 10%, and 2%, respectively. Yentür et al. [25] investigated the contamination of 20 peanut butter samples with aflatoxins by the HPLC method, and they found the mean values of aflatoxins B1, B2, and G1 as 15,756 ± 3.129 ng/g, 1.232 ± 0.244 ng/g, and 9.689 ± 1.005 ng/g, respectively. Ren et al. [26] analyzed mycotoxins in foods and feeds by ultra-performance liquid chromatography–tandem mass spectrometry. They have stated that the mean values of AFB1, AFB2, AFG1, AFG2, and OTA in peanut butter are between 0.41–31.44 µg/kg, 0.01–7.47 µg/kg, 0.06–16.50 µg/kg, 0.06–1.54 µg/kg, and 0.24–0.42 µg/kg, respectively. They reported that ZON and DON mycotoxins were detected as 3.95 µg/kg and 1.04 µg/kg in one sample, respectively, and T2 and HT2 toxins were not detected in the samples. Kumagai et al. [27] analyzed aflatoxin B1, B2, G1, G2, and ochratoxin A contamination in various foods. They stated that they detected aflatoxin in 10 of 21 peanut butter samples and 22 of 44 dark chocolate samples, and the highest aflatoxin B1 value was detected in peanut butter with 2.59 µg/kg. They reported that the mean values of aflatoxin B1, B2, G1, and G2 in peanut butter samples were 1.07 µg/kg, 0.27 µg/kg, 0.4 µg/kg, and 0.21 µg/kg, respectively. They stated that the average of aflatoxin B1 was 0.18 µg/kg in dark chocolate, but they did not detect aflatoxin B2, G1, and G2. They also stated that the average value of ochratoxin A in dark chocolate is 0.35 µg/kg. Leong et al. [28] reported that the levels of aflatoxin B1 and aflatoxin B2 in 7 (58.3%) of 12 peanut butter samples were between 13.3–56.6 µg/kg and 3.31–10.8 µg/kg, respectively. Elzupir et al. [29] investigated the amount of aflatoxins in 43 peanut butter samples and found AFB1 as 223 ± 124.2 µg/kg in 28 samples, AFG1 as 137 ± 77.9 µg/kg in 43 samples, AFB2 as 3.20 ± 4.4 µg/kg in 42 samples, and AFG2 as 18.5 ± 9.5 µg/kg in 4 samples. Chen et al. [30] investigated aflatoxin levels in 1827 commercial peanut products in Taiwan between 1997 and 2011. It was stated that the samples were analyzed in terms of aflatoxin B1, B2, G1, and G2 by high-performance liquid chromatography, and Aflatoxins were detected in 32.7% of the samples at levels ranging from 0.2 µg/kg to 513.4 µg/kg. They reported that peanut butter was the product with the highest incidence of aflatoxin, total aflatoxin was 2.8 µg/kg in 75 (52.18%) of 142 peanut butter samples, and mean values of AFB1 and AFB2 were 1.27 µg/kg and 0.40 µg/kg, respectively. They stated that aflatoxin B1 had the highest detection frequency among aflatoxin positive samples, followed by aflatoxin B2, aflatoxin G2, and aflatoxin G1. Mupunga et al. [31] analyzed the contamination of aflatoxins by the HPLC method in 11 peanut butter samples in Bulawayo, Zimbabwe. They reported that the ranges of aflatoxin B1, B2, G1, and G2 in peanut butter are 6.1–191 µg/kg, ND–25.7 µg/kg, ND–47.1 µg/kg, and ND–8.8 µg/kg, respectively. Keskin and Gürsoy [32] investigated the formation of aflatoxin in hazelnut products and stated that 9 out of 20 hazelnut paste samples had aflatoxin contamination between 0.2 µg/kg and 6.02 µg/kg.

Classical culture techniques are generally used to detect and count living microorganisms in the microbiological analysis of foods [33]. The total aerobic mesophilic plate count, often referred to as aerobic plate count or standard plate count, is the most commonly used assay to provide information about bacterial populations in foods. The use of total aerobic mesophilic plaque count as a quality indicator should be performed with caution [34]. Odu and Okonko [35] investigated the microbiological quality of traditionally processed peanut butter sold in the metropolitan Port Harcourt, Rivers State, Nigeria, and reported that the total viable microorganism count was between 3.5 × 10^2^ cfu/g and 2.3 × 10^3^ cfu/g. El-Sisy and Ali [36] found that, in their study, the total number of aerobic bacteria in peanut butter is 1.91 log cfu/g on the use of nutrient-rich cereal pastes as an alternative to peanut butter.

## 4. Materials and Methods

### 4.1. Samples Collection

A total of 80 chocolate (A and B brands), hazelnut butter (C and D brands), and peanut butter (E, F, G, and H brands) were obtained from various local markets in Ankara, Turkey. The samples were not expired and each had a different serial number and were collected between April and July 2021. Brands of the collected nut and chocolate samples were also sold all over Turkey.

### 4.2. Sample Preparation

The instructions of the Jasem LC–MS/MS multi-mycotoxin analysis kit (SEM, İstanbul-Turkey) were considered for the preparation of the samples for analysis [37]. For this purpose, hazelnut, peanut butter, and chocolate samples were homogenized, and a 5.0 g sample was transferred to a 50 mL centrifuge tube. Then, 20 mL of reagent 1 (Jasem, JSM FO 9704, SEM, İstanbul-Turkey) was added to each sample before mixing the samples with the multi-shaker for 15 min. After shaking, the solution in the tube was centrifuged at 3000 rpm for 5 min at room temperature. The clear supernatant of the samples after centrifugation was filtered into HPLC vials using 0.45 micron nylon filters and injected directly into the LC–MS/MS instrument.

### 4.3. LC–MS/MS Procedure

**Figure 1:**
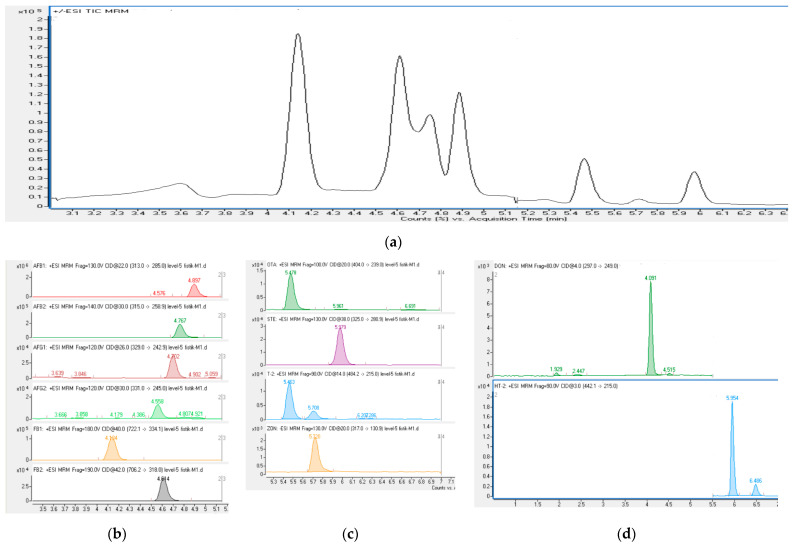
LC−MS/MS chromatograms and TIC of mycotoxins: (**a**) TIC. (**b**) AFB1; aflatoxin B1, AFB2; aflatoxin B2, AFG1; aflatoxin G1, AFG2; aflatoxin G2, FB1; fumonisin B1, FB2; fumonisin B2. (**c**) OTA; ochratoxin A, STE; sterigmatocystin, T2; T-2 toxin, ZON; zearalenone. (**d**) DON; deoxynivalenol and HT2; HT-2 toxin.

A commercial multi-mycotoxin LC–MS/MS kit was used for multi-mycotoxin (aflatoxin B1, B2, G1, and G2; fumonisin B1 and B2; ochratoxin A; sterigmatocystin; deoxynivalenol; zearalenone; T-2; and HT-2 toxin) analysis in hazelnut and peanut butter and chocolate samples, and the manufacturer’s instructions were followed in the application of the analysis [37]. Mobile phases, sample preparation reagent, calibration standards, and analytical column were from the ready-to-use Jasem multi-mycotoxins kit (SEM, İstanbul-Turkey). In the analysis of multiple mycotoxins, an Agilent LC 1290 combination with a 6470 triple quadrupole mass spectrometer (LC–MS/MS), electrospray ionization (ESI) (Agilent Technologies, Santa Clara, CA, USA), and a Jasem analytical column were used (JSM-FO-9775, SEM, İstanbul-Turkey). Gradient elution was performed using mobile phase A (JSM-FO-9701), mobile phase B (JSM-FO-9702), mobile phase C (JSM-FO-9703), and gradient: 0–1 min 20% B, 1–4 min 2–95% B, 4–7 min 95% B, 7–7.1 min 20% B, 7.1–12 min 20% B, MS detection for AFB1, AFB2, AFG1, AFG2, FB1, FB2, STE, T2, ZON, and OTA. In HT2 and DON mycotoxins, gradient: 0–1 min 20% C, 1–4 min 2–95% C, 4–7 min 95% C, 7–7.1 min 20% C, 7.1–12 min 20% C, MS detection. The column furnace temperature was set to 35 °C. The flow rate was 0.5 mL/min and the injection volume was 10 µL. The AJS ESI ion source parameters used in the mass spectrometer are as follows: gas temperature 300 °C, nebulizer gas (N_2_) flow 11 L/min, nebulizer pressure 40 (psi), sheath gas temperature 400 °C, sheath gas (N_2_) flow 11 L/min, as well as capillary voltage positive +3500 V and negative −3500 V. A MassHunter 647 Workstation (Version 10.1, Agilent Technologies, Santa Clara, CA, USA) was used for data collection, method creation, and qualitative and quantitative analysis. As a result of the analysis, the levels of mycotoxins in the samples were expressed as μg/kg. For the quantitative analysis of the samples, multiple reaction monitoring (MRM) was run in scanning type. Furthermore, molecular ions (precursor ions) were used as one or two degradation products (fragment ions)—the first of which was obtained for quantitative analysis and the second for qualitative analysis. LC–MS/MS chromatograms of twelve mycotoxins are shown in Figure 1.

Validation parameters assessed for nut butter (representative peanut) and chocolate were correlation coefficient (R2), relative standard deviation (RSD), the limit of detection (LOD), the limit of quantification (LOQ), and recovery (Table 6).

Parent ion and fragment ions, retention time, concentration ranges, fragmentor voltage, and collision energies of the mycotoxins are presented in the Table 7.

### 4.4. Total Aerobic Mesophilic Bacteria (TAMB) Analysis

Total aerobic mesophilic bacteria (TAMB) counts of the samples were determined by the classical culture method. Ten grams of hazelnut and peanut butter and chocolate samples were weighed aseptically. Then, 90 mL of maximum recovery diluent (MRD, Merck 1.12535, Darmstadt, Germany) was added to the sample, and the sample was homogenized using a Stomacher laboratory mixer (Bagmixer 400, aint-Nom-la-Bretèche, France). Serial dilutions were created by taking 1 mL of this homogeneous sample and diluting it with 9 mL of MRD. Inoculations were made using the spread plate method from three serial dilutions on Plate Count Agar (PCA, Merck 1.05463, Darmstadt, Germany). Post-inoculation PCA plates were incubated at 37 °C for 24 h. Colonies growing on the plates at the end of the incubation period were counted and expressed as log colony-forming units (log CFU/g) per gram sample [38].

### 4.5. Statistical Analyses

Levene’s test was applied to determine the homogeneity of variances of the groups. The normal distribution of the groups was checked with the Shapiro–Wilk Test. When the data of the groups showed normal distribution, the independent samples t-test was applied to statistically evaluate the difference between the two groups. The Mann–Whitney U-Test was used to compare these two groups in cases where one or both of the two different groups did not satisfy the assumption of normality. The one-way ANOVA test was used to compare the data of more than two groups. The Bonferroni test was used in the comparison of the difference between the groups in those who showed homogeneous variance distribution, and the Tamhane T2 test was used in those who did not show homogeneous variance distribution [39].

## 5. Conclusions

AFG2, FB1, T2, DON, and HT2 toxins were not detected in peanut butter. Considering the mycotoxin prevalence in peanut butter, AFB1 and AFB2 were detected in 100% of the samples. OTA, STE, FB2, ZON, and AFG1 mycotoxins in peanut butter were determined as 95%, 45%, 12.5%, 7.5%, and 5%, respectively. AFG2, FB1, FB2, and DON toxins were not detected in hazelnut butter. AFB1, AFG1, AFB2, T2, OTA, ZON, HT2, and STE mycotoxins in hazelnut butters were determined as 50%, 35%, 30%, 30%, 25%, 25%, 20%, and 15%, respectively. AFB2, AFG2, FB1, FB2, T2, and HT2 toxins were not detected in chocolate samples. OTA mycotoxin was detected at the rate of 100% in chocolate samples. DON, AFB1, AFG1, ZON, and STE mycotoxins were determined as 55%, 50%, 50%, 50%, and 40% in chocolate samples, respectively. Mycotoxins were detected in the range of 0.01 to 42.09 µg/kg in the analyzed 80 commercial food samples from Ankara. The number of samples exceeding the maximum value specified in the Turkish Food Codex (TFC) in terms of AFB1 and total aflatoxin amounts in peanut butter was determined as 21 (52.5%) and 12 (30%), respectively. Consumption of foods at risk for mycotoxins (foods containing mycotoxins above the limits specified in the TFC) by particularly sensitive individuals is an important issue affecting public health. For this reason, systematic monitoring of risky foods is very important in ensuring food safety and protecting public health.

## Figures and Tables

**Table 1:** Mycotoxin mean ± S.E, as well as the min and max data of positive chocolate samples.

Mycotoxins		Chocolate Brands	
		A	B
AFB1	N (Positive/Total)	10/10	nd
	Mean ± S.E (μg/kg)	0.18 ± 0.02	nd
	Min (μg/kg)	0.11	nd
	Max (μg/kg)	0.24	nd
AFG1	N (Positive/Total)	10/10	nd
	Mean ± S.E (μg/kg)	0.23 ± 0.01	nd
	Min (μg/kg)	0.17	nd
	Max (μg/kg)	0.27	nd
OTA	N (Positive/Total)	10/10	10/10
	Mean ± S.E (μg/kg)	1.87 ^a^ ± 0.11	0.44 ^b^ ± 0.07
	Min (μg/kg)	1.20	0.13
	Max (μg/kg)	2.34	0.68
STE	N (Positive/Total)	8/10	nd
	Mean ± S.E (μg/kg)	1.01 ± 0.14	nd
	Min (μg/kg)	0.49	nd
	Max (μg/kg)	1.50	nd
ZON	N (Positive/Total)	10/10	nd
	Mean ± S.E (μg/kg)	3.11 ± 0.64	nd
	Min (μg/kg)	0.90	nd
	Max (μg/kg)	7.57	nd
DON	N (Positive/Total)	1/10	10/10
	Mean ± S.E (μg/kg)	29.52	29.81 ± 2.77
	Min (μg/kg)	29.52	11.81
	Max (μg/kg)	29.52	42.09

nd: not detected. ^a,b^: Mean values with different letters in the same row are statistically different (*p* < 0.05). AFB1: aflatoxin B1, AFG1: aflatoxin G1, OTA: ochratoxin A, STE: sterigmatocystin, ZON: zearalenone, and DON: deoxynivalenol.

**Table 2:** Mycotoxin mean ± S.E, as well as the min and max data of positive hazelnut butter samples.

Mycotoxins		Hazelnut Butters	
		C	D
AFB1	N (Positive/Total)	4/10	6/10
	Mean ± S.E (μg/kg)	1.03 ± 0.72	0.21 ± 0.07
	Min (μg/kg)	0.27	0.04
	Max (μg/kg)	3.18	0.49
AFB2	N (Positive/Total)	4/10	2/10
	Mean ± S.E (μg/kg)	0.12 ± 0.03	0.08 ± 0.01
	Min (μg/kg)	0.06	0.07
	Max (μg/kg)	0.20	0.08
AFG1	N (Positive/Total)	2/10	5/10
	Mean ± S.E (μg/kg)	0.17 ± 0.005	0.22 ± 0.05
	Min (μg/kg)	0.16	0.14
	Max (μg/kg)	0.17	0.40
OTA	N (Positive/Total)	2/10	3/10
	Mean ± S.E (μg/kg)	0.59 ± 0.35	0.05 ± 0.02
	Min (μg/kg)	0.24	0.01
	Max (μg/kg)	0.94	0.08
STE	N (Positive/Total)	1/10	2/10
	Mean ± S.E (μg/kg)	0.23	0.66 ± 0.44
	Min (μg/kg)	0.23	0.22
	Max (μg/kg)	0.23	1.09
T2	N (Positive/Total)	3/10	3/10
	Mean ± S.E (μg/kg)	1.75 ± 0.19	2.09 ± 0.23
	Min (μg/kg)	1.41	1.77
	Max (μg/kg)	2.08	2.54
ZON	N (Positive/Total)	2/10	3/10
	Mean ± S.E (μg/kg)	2.41 ± 1.14	0.98 ± 0.48
	Min (μg/kg)	1.27	0.33
	Max (μg/kg)	3.55	1.91
HT2	N (Positive/Total)	2/10	2/10
	Mean ± S.E (μg/kg)	7.01 ^b^ ± 0.07	7.42 ^a^ ± 0.02
	Min (μg/kg)	6.94	7.40
	Max (μg/kg)	7.07	7.43

^a,b^: Mean values with different letters in the same row are statistically different (*p* < 0.05). AFB1: aflatoxin B1, AFB2: aflatoxin B2, AFG1: aflatoxin G1, OTA: ochratoxin A, STE: sterigmatocystin, T2: T-2 toxin, ZON: zearalenone, and HT2: HT-2 toxin.

**Table 3:** Mycotoxin mean ± S.E, as well as the min and max data of positive peanut butter samples.

Mycotoxins		Peanut Butter Brands			
		E	F	G	H
AFB1	N (Positive/Total)	10/10	10/10	10/10	10/10
	Mean ± S.E (μg/kg)	5.37 ± 2.20	13.35 ^a^ ± 2.37	8.11 ^b^ ± 1.56	0.94 ^c^ ± 0.24
	Min (μg/kg)	0.18	5.56	1.02	0.20
	Max (μg/kg)	22.97	27.37	15.67	2.89
AFB2	N (Positive/Total)	10/10	10/10	10/10	10/10
	Mean ± S.E (μg/kg)	0.93 ± 0.36	2.53 ^a^ ± 0.60	1.54 ^b^ ± 0.28	0.20 ^c^ ± 0.03
	Min (μg/kg)	0.08	0.87	0.28	0.10
	Max (μg/kg)	3.86	6.19	3.02	0.46
AFG1	N (Positive/Total)	nd	2/10	nd	nd
	Mean ± S.E (μg/kg)	nd	0.21 ± 0.03	nd	nd
	Min (μg/kg)	nd	0.18	nd	nd
	Max (μg/kg)	nd	0.24	nd	nd
FB2	N (Positive/Total)	nd	nd	5/10	nd
	Mean ± S.E (μg/kg)	nd	nd	2.85 ± 0.04	nd
	Min (μg/kg)	nd	nd	2.73	nd
	Max (μg/kg)	nd	nd	2.93	nd
OTA	N (Positive/Total)	10/10	10/10	10/10	8/10
	Mean ± S.E (μg/kg)	2.87 ± 1.85	7.02 ± 3.39	4.43 ± 0.92	0.17 ± 0.05
	Min (μg/kg)	0.19	1.72	0.51	0.09
	Max (μg/kg)	19.20	37.26	9.26	0.47
STE	N (Positive/Total)	5/10	10/10	3/10	0/10
	Mean ± S.E (μg/kg)	0.40 ^b^ ± 0.11	1.23 ^a^ ± 0.19	0.57 ± 0.17	nd
	Min (μg/kg)	0.19	0.47	0.26	nd
	Max (μg/kg)	0.83	2.25	0.86	nd
ZON	N (Positive/Total)	nd	2/10	1/10	nd
	Mean ± S.E (μg/kg)	nd	0.08 ± 0.05	0.24	nd
	Min (μg/kg)	nd	0.03	0.24	nd
	Max (μg/kg)	nd	0.12	0.24	nd

nd: not detected. ^a,b^: Mean values with different letters in the same row are statistically different (*p* < 0.05). ^a,b,c^: Mean values with different letters in the same row are statistically different (*p* < 0.05). AFB1: aflatoxin B1, AFB2: aflatoxin B2, AFG1: aflatoxin G1, FB2: fumonisin B2, OTA: ochratoxin A, STE: sterigmatocystin, and ZON: zearalenone.

**Table 4:** Mean ± S.E TAMB values of positive samples of chocolates and nut butter.

TAMB	Positive Samples (%)	Number of Positive Samples	Mean ± S.E (log CFU/g)
Chocolates	50	10	2.41 ± 0.08
Hazelnut butters	5	1	2.73
Peanut butters	80	32	2.74 ± 0.06
Total	53.75	43	2.66 ± 0.05

TAMB: total aerobic mesophilic bacteria.

**Table 5:** Mean ± S.E, as well as the min and max TAMB values of brands of chocolates, hazelnut, and peanut butter.

**TAMB**	**Peanut Butters**			
	**E**	**F**	**G**	**H**
N (Positive/Total)	3/10	10/10	10/10	9/10
Mean ± S.E (log CFU/g)	2.23 ^c^ ± 0.04	2.90 ^a^ ± 0.09	2.89 ^b^ ± 0.10	2.57 ± 0.10
Min (log CFU/g)	2.15	2.54	2.48	2.15
Max (log CFU/g)	2.30	3.43	3.59	3.12
**TAMB**	**Chocolates**		**Nut Butters**	
	**A**	**B**	**C**	**D**
N (Positive/Total)	7/10	3/10	0/10	1/10
Mean ± S.E (log CFU/g)	2.52 ^a^ ± 0.07	2.15 ^b^ ± 0.00	<10^2^	2.73
Min (log CFU/g)	2.24	2.15	<10^2^	2.73
Max (log CFU/g)	2.77	2.15	<10^2^	2.73

^a,b^: mean values with different letters in the same row are statistically different (*p* < 0.05). ^a,b,c^: mean values with different letters in the same row are statistically different (*p* < 0.05). TAMB: total aerobic mesophilic bacteria.

**Table 6:** Validation parameters of the LC–MS/MS method applied for nut butter and chocolate.

Mycotoxins	Nut Butter					Chocolate				
	RSD (%)	Rec (%)	LOD (µg/kg)	LOQ (µg/kg)	R^2^	RSD (%)	Rec (%)	LOD (µg/kg)	LOQ (µg/kg)	R^2^
AFB1	3.84	96.15	0.03	0.09	0.997	2.95	98.38	0.02	0.18	0.998
AFB2	3.23	94.62	0.02	0.08	0.997	3.94	88.92	0.03	0.32	0.998
AFG1	2.44	95.15	0.02	0.06	0.998	4.87	101.88	0.02	0.18	0.993
AFG2	11.24	101.55	0.09	0.29	0.993	3.51	101.55	0.01	0.11	0.995
DON	4.62	110.68	3.83	12.77	0.999	4.24	102.69	2.38	23.77	0.992
FB1	3.81	74.13	2.12	7.07	0.999	5.22	53.18	0.65	6.51	0.998
FB2	2.33	83.89	1.47	4.89	0.998	1.79	76.75	0.19	1.88	0.998
HT2	3.19	111.40	2.66	8.88	0.999	12.63	86.54	1.43	14.31	0.997
OTA	6.32	95.20	0.01	0.15	0.995	4.3	98.91	0.02	0.19	0.997
STE	2.29	94.16	0.02	0.05	0.994	3.53	100.04	0.01	0.15	0.998
T2	2.96	95.55	0.21	0.71	0.994	2.98	95.52	0.14	1.38	0.998
ZON	4.40	94.78	0.03	0.11	0.996	1.78	102.33	0.11	1.12	0.999

AFB1: aflatoxin B1, AFB2: aflatoxin B2, AFG1: aflatoxin G1, AFG2: aflatoxin G2, DON: deoxynivalenol, FB1: fumonisin B1, FB2: fumonisin B2, HT2: HT-2 toxin, OTA: ochratoxin A, STE: sterigmatocystin, T2: T-2 toxin and ZON: zearalenone.

**Table 7:** Optimized MS/MS parameters for the mycotoxins.

Mycotoxins	RT	Parent Ion (m/z)	Fragment Ions	Concentration Range (µg/L)	Ion Mode	Fragmentor Voltage (V)	CE (V)	
AFB1	4.89 ± 0.01	313	285/240.9	0.05–1	Positive	130	22	38
AFB2	4.76 ± 0.01	315	287/258.9	0.05–1	Positive	140	24	30
AFG1	4.70 ± 0.03	329	242.9/200	0.05–1	Positive	120	26	38
AFG2	4.55 ± 0.02	331	312.9/245	0.05–1	Positive	120	24	30
DON	4.09 ± 0.05	297	249/231	5–100	Positive	80	4	6
FB1	4.13 ± 0.05	722.1	352.1/334.1	5–100	Positive	180	36	40
FB2	4.61 ± 0.01	706.2	336.1/318	1.5–30	Positive	190	40	42
HT2	5.95 ± 0.01	442.1	263/215	5–100	Positive	90	3	3
OTA	5.47 ± 0.01	404	358/239	0.05–1	Positive	100	10	20
STE	5.97 ± 0.02	325	309.9/280.9	0.05–1	Positive	130	24	38
T2	5.46 ± 0.01	484.2	215/185	0.5–10	Positive	90	14	16
ZON	5.72 ± 0.01	317	174.9/130.9	0.5–10	Negative	130	12	20

RT: retention time, CE: collision energies.

## Data Availability

The datasets analyzed during the study are included in the article.
